# How does the TRS 2°P score relate to real-world patients?

**DOI:** 10.1093/ehjcvp/pvy004

**Published:** 2018-03-05

**Authors:** Marc P Bonaca, Gaetano M De Ferrari, Dan Atar, Lori D Bash, Dominik Lautsch, Erin A Bohula, Martin Horack, Philippe Brudi, Jean Ferrieres, Anselm K Gitt

**Affiliations:** 1 TIMI Study Group, Cardiovascular Division, Brigham and Women’s Hospital, Boston, MA, USA; 2 Department of Molecular Medicine University of Pavia, and Cardiac Intensive Care Unit and Laboratories for Experimental Cardiology, IRCCS Fondazione Policlinico San Matteo, Pavia, Italy; 3 Oslo University Hospital, Department of Cardiology B, and Institute of Clinical Sciences, University of Oslo, Norway; 4 Merck&Co., Inc., Kenilworth, NJ, USA; 5 Stiftung Institut für Herzinfarktforschung Ludwigshafen, Ludwigshafen, Germany; 6 Department of Cardiology, Toulouse Rangueil Hospital, Toulouse University School of Medicine, Toulouse, France; 7 Medizinische Klinik B, Klinikum der Stadt Ludwigshafen, Ludwigshafen, Germany

Patients suffering from atherosclerotic cardiovascular disease (ASCVD) are at heightened risk for future atherothrombotic events, which are a leading cause of mortality worldwide.^1^ Patients who suffer an acute coronary syndrome (ACS) are at further increased risk relative to those with stable disease and no history of ACS. In this context, the recently derived and validated TIMI Risk Score for Secondary Prevention (TRS 2°P) is a novel tool for better determiniation of an individual patient’s risk for major adverse cardiovascular events (MACE) after ACS.^2^ Developed with the use of data from a large randomized controlled trial (RCT), TRA 2°P-TIMI-50,^2^ the TRS 2°P score is calculated based on the presence of nine risk factors, with each contributing a point (*Table 1*).^2^ The TRS 2°P was recently applied in the IMPROVE-IT RCT,^3^ where patients stabilized post-ACS were randomized to treatment with the cholesterol-lowering agent ezetimibe, or matching placebo, on a background of statin therapy.^4^ Overall, the addition of ezetimibe to simvastatin resulted in an achieved LDL-cholesterol (LDL-C) of 53.2 mg/dL compared with 69.9 mg/dL with simvastatin alone and, importantly, lower rates of cardiovascular events. Application of the TRS 2°P in IMPROVE-IT identified post-ACS patients at the highest risk for recurrent cardiovascular events who also derived the greatest absolute risk reduction with ezetimibe.

**Table 1:** Characteristics of DYSIS II CHD patients (*n* = 5371)

	Mean ± SD or %
Age (years)	65.6 ± 10.8
Male	78.5
TRS 2°P component	
Congestive heart failure	12.3
Hypertension	96.0
Age ≥75 years	22.0
Diabetes mellitus	40.3
Prior stroke	5.4
Prior CABG	22.3
Peripheral artery disease	9.7
eGFR <60 mL/min/1.73 m^2^	20.1
Current smoking	12.6

CABG, coronary artery bypass graft; eGFR, estimated glomerular filtration rate.

While RCT data are the foundation of clinical evidence when determining the therapeutic efficacy of drugs, patients are inherently highly selected and may represent the younger, healthier individuals among the disease population. In addition, if broad application of preventive strategies such as ezetimibe are not possible due to resource constraints, identifying subgroups of post-ACS patients who are at even higher risk and therefore derive greater benefit from secondary preventative therapies may be useful. We therefore wished to assess the distribution of risk in a real-world population of patients with ASCVD as defined by the TRS 2°P. In addition, we evaluated the use of high intensity lipid-lowering therapy and achieved LDL-C in these high-risk groups to assess whether patterns of treatment were associated with patient risk.

The DYSIS II CHD study was a cross-sectional observational study of 6794 patients with stable coronary heart disease (CHD) in 18 countries across the globe.^5^ The study complies with the Declaration of Helsinki and the research protocol was approved by ethics committees according to local regulations. All patients provided written informed consent. Patients who were ≥18 years old were enrolled if they were attending an outpatient physician appointment for stable CHD between 2012 and 2014, and had a full lipid profile available from within the previous 12 months. For risk stratification using the TRS 2°P, a full data set was available for 5371 patients. Of the nine components of the TRS 2°P, hypertension (96.0%) and diabetes mellitus (40.3%) were the most commonly found in the DYSIS II population (*Table 1*). Only 2.0% of patients had a TRS 2°P score of zero, while 3.7% had a score of ≥5 (*Figure 1*). When compared with the patients enrolled in the IMPROVE-IT RCT, the DYSIS II CHD population displayed a greater number of TRS 2°P risk factors, with 12% of the patients in the IMPROVE-IT control arm (simvastatin treatment) having a score of zero and 2% having a score of ≥5.^3^ This higher risk profile among an unselected real-world population compared with the RCT population was expected and is confirmed by our findings.

**Figure 1:**
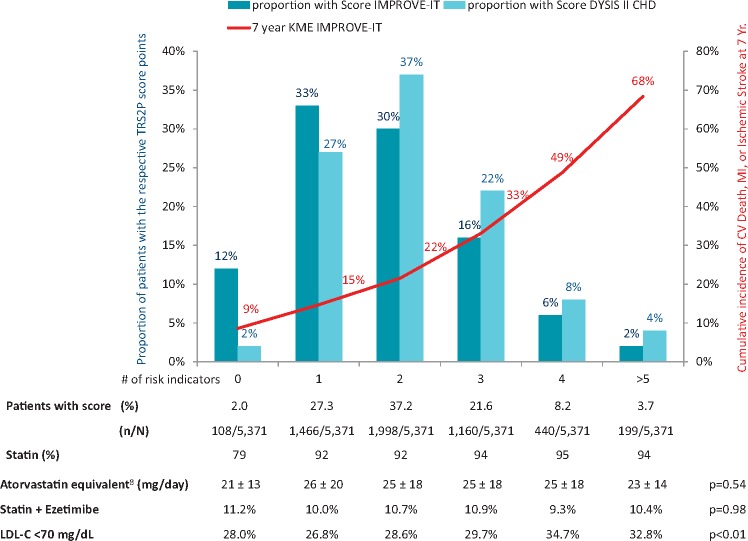
Distribution of TRS 2°P risk categories in IMPROVE-IT and DYSIS II CHD; LDL-C target attainment and lipid-lowering therapy according to TRS 2°P. Numbers below the graph refer to DYSIS II CHD; *P*-values were calculated using the Cochran–Armitage test for trend statin + ezetimibe combination and LDL-C <70 mg/dL (equal to <1.8 mmol/L), and the Jonckheere–Terpstra test for trend atorvastatin equivalents. LDL-C, LDL-cholesterol; KME, Kaplan–Meier estimates referring to the simvastatin-treated control group in IMPROVE-IT; CV, cardiovascular; Yr, year.

Although cardiovascular events over time were not captured in the DYSIS II CHD study, by extrapolating the risk of events in IMPROVE-IT, it would be expected that the event rate would have been higher for the DYSIS II patients. The cumulative incidence of MACE for the simvastatin-treated patients in IMPROVE-IT who had a TRS 2°P of ≥5 was as high as 68.4% during the 7 years of follow-up; however, the simvastatin/ezetimibe combination lowered the risk of an event.^3^ The absolute benefits of ezetimibe addition to the statin therapy were most pronounced in the patients with higher TRS 2°P values, which indicates that use of ezetimibe would have even greater benefits for the higher risk DYSIS II CHD population. However, use of lipid-lowering combination therapy in DYSIS II CHD was low, at ∼10% (*Figure 1*). Furthermore, its use did not vary depending on the number of TRS 2°P risk indicators that patients had.

These findings suggest that current approaches to reducing LDL-C in patients with ASCVD and particularly post-ACS may not sufficiently target the highest risk patients with the most intensive therapies. Many of the expert-driven guidelines recommend LDL-C targets for patients that depend on their level of cardiovascular risk, with <70 mg/dL (<1.8 mmol/L) often the goal for those at highest risk.^6^^,^^7^ In DYSIS II CHD, all patients were classified as being at very high risk according to European guidelines,^6^ but attainment of the <70 mg/dL target was poor. Despite the majority of subjects being treated with a statin, the mean atorvastatin-equivalent daily statin dosage^8^ was low for all of the TRS 2°P risk groups. This, in combination with the universally low use of ezetimibe, suggests a high level of undertreatment regardless of patient risk profile.

A more personalized approach to preventive therapies focused on an individual patient’s risk, rather than their lipid level, may be an alternative way to target therapies in broad populations. As increasing evidence shows that lowering LDL-C to very low levels is safe and reduces risk,^10^ the use of LDL-C targets may be less relevant and the question for clinicians will be to identify which patients should receive the most intensive strategies. While lipid levels should not be overlooked, maximal intensive treatment should be considered for all patients with high TRS 2°P scores, to achieve an LDL-C as low as possible. This approach is more similar to that stated in the most recent guidelines from the USA and Canada, where the presence of ASCVD is always considered an indication for lipid-lowering treatment.^9^^,^^11^ Use of the TRS 2°P in clinical practice could further refine treatment recommendations, by simple stratification of patients with CHD according to the presence of nine specific co-morbidities. The TRS2°P risk score includes six risk factors also contained in the CHA_2_DS_2_-VASc, widely used in assessing cardiovascular risk in atrial fibrillation patients (congestive heart failure, hypertension, age ≥75 years, prevalence of diabetes mellitus, prior stroke, and vascular disease, including peripheral artery disease),^12–14^ adding three additional clinical features (prior coronary artery bypass graft, moderate renal insufficiency, and current smoking). All of these factors were found to have similar weight in the prediction of MACE in post-ACS patients.^2^ Further validation of TRS 2°P, and its adoption into clinical practice, may help to better tailor the treatment of the individual patient with CHD contributing to a reduction in the incidence of MACE.

## Funding

This work was supported by Merck & Co., Inc., Kenilworth, NJ, USA.

**Conflict of interest:** M.P.B. received a speaker’s fee from Merck & Co. Inc., Kenilworth, NJ, USA. G.M.D.F. reports grants and personal fees from MSD during the conduct of the study; grants and personal fees from Amgen; grants from Boston Scientific; and personal fees from SigmaTau outside the submitted work. D.A. received speakers’ and consultancy honoraria from MSD, Amgen, Sanofi, and Pfizer. L.D.B., D.L., and P.B. are employees of Merck & Co. Inc., Kenilworth, NJ, USA. E.B. reports advisory honoraria from Merck during the conduct of the study; personal fees from Servier for consulting; and for an advisory committee from Novartis, outside the submitted work. M.H. has nothing to disclose. J.F. reports grants and personal fees from Amgen, and personal fees from MSD and Sanofi during the conduct of the study. A.K.G. received honoraria from MSD for contributions to DYSIS II CHD.
